# Anti-Interference Detection of *Vibrio parahaemolyticus* from Aquatic Food Based on Target-Cyclized RCA with Dynamic Adapter Followed by LAMP

**DOI:** 10.3390/foods11030352

**Published:** 2022-01-26

**Authors:** Boying Zhang, Wenhua Sun, Lingling Ran, Chenru Wang, Jing Wang, Ran An, Xingguo Liang

**Affiliations:** 1 College of Food Science and Engineering, Ocean University of China, Qingdao 266003, China; chloe0114@163.com (B.Z.); sunwh0702@163.com (W.S.); MargheritaRoo@163.com (L.R.); mariahcareysuper@163.com (C.W.); wodejia_sun@126.com (J.W.); liangxg@ouc.edu.cn (X.L.); 2 Laboratory for Marine Drugs and Bioproducts, Qingdao National Laboratory for Marine Science and Technology, Qingdao 266235, China

**Keywords:** biological contaminants, foodborne pathogens, *Vibrio parahaemolyticus*, targeted detection, isothermal amplification

## Abstract

*Vibrio parahaemolyticus* (*V. parahaemolyticus*) is considered the most concerning pathogen for seafood. Like other pathogens in food samples, its gene detection suffers from a problem of background interference when isothermal detection methods are used. The sensitivity and specificity greatly decrease due to large amounts of background genome. Here we describe a novel isothermal detection technology based on target-cyclized rolling circle amplification combined with loop-mediated isothermal amplification (tRCA-lamp). By avoiding unexpected ligation, a short dynamic adapter is employed to increase the sensitivity of target cyclization in the presence of the background genome. At the amplification step, highly specific detection is obtained by linear RCA and simplified LAMP (only two primers are used). Furthermore, visual detection is easily realized with hydroxynaphthol blue (HNB). In the oyster samples, the tRCA-lamp approach can detect *V. parahaemolyticus* with a detection limit of 22 cfu/g with none necessary to enrich the bacteria and remove the host DNA. This method gets rid of the complicated primer design process and can be extended to the detection of other pathogens in food samples.

## 1. Introduction

*Vibrio parahaemolyticus* (*V. parahaemolyticus*) is a type of pathogenic bacteria that is commonly found in aquatic foods, including shellfish and shrimp [1]. It grows rapidly in 3~5% NaCl and can tolerate high salt (up to 10%) and low-temperature environments (lower than 15 °C) [2]. *V. parahaemolyticus* is the causative agent causing food poisonings, such as diarrhea, nausea, vomiting, abdominal cramps, and even death [3]. Although high temperatures can kill them [4], raw seafood is easily contaminated with this pathogen and can cause serious food poisoning cases because more and more people like to eat them directly [5]. Therefore, highly efficient detection methods are required to avoid food poisoning by this pathogen.

For raw foods whose shelf lives are quite short, the rapid detection method is more suitable for the screening of large numbers of samples compared with traditional culture methods [6]. The two major categories of rapid approaches for detecting *V. parahaemolyticus* is the immunological method [7] and nucleic acid detection [8]. Nucleic acid detection offers direct evidence for the presence of the pathogen in samples [9]. At present, many researchers use real-time PCR to detect *V. parahaemolyticus* in aquatic food samples, and obtain high sensitivity and specificity [10,11]. However, for isothermal methods that do not require temperature changing equipment and are more convenient (e.g., RCA and LAMP), the bacteria are usually separated from the food samples by culture or aptamer capture before the detection [12,13]. One possible reason is that isothermal amplification is easily interfered with by the background genome of seafood, which usually has much longer genome DNA [14]. For example, if there are 100 cfu bacterium in a 25 g oyster, the weight of the background genome from the oyster is 10^10^ times more than that of the pathogen [15]. Such a large amount of host DNA may contain similar sequences with the target, resulting in the misrecognition of primers. Compared with annealing hybridization in PCR, the hybridization of primers in isothermal amplification is more susceptible to these similar sequences. To avoid non-specific amplification, some studies designed multiple pairs of primers separately targeting multiple genes, which is a non-generic method for different pathogens and requires a complicated primer design process [16].

Recently, we developed a mode of anti-interference RCA, designated as target-cyclization rolling circle amplification (TC-RCA). In this approach, a double-stranded DNA target is directly served as the template of amplification after being cyclized by a double-stranded adapter [17]. As compared with the padlock-RCA [18], target DNA has learned not to denature, and the specificity has been improved significantly because the primers directly targeted the *V. parahaemolyticus* gene. However, its sensitivity is unsatisfactory due to the low target cyclization yield. Herein, we present a novel rapid anti-interference detection method with much higher sensitivity. In the present approach, the unexpected ligation blocking target cyclization is greatly avoided by using a short dynamic adapter in order to satisfactorily improve the cyclization sensitivity. In the following step, highly specific amplification of the cyclized target is carried out by combining linear RCA and simplified LAMP, which are carried out simultaneously. The linear RCA is employed to pre-amplify the target from a large amount of background DNA, and the single-stranded products with long repeated sequences act as the template of LAMP in which only one pair of primers targeting *V. parahaemolyticus* gene is used. Furthermore, visual detection is easily realized with hydroxynaphthol blue (HNB). The combination of target-cyclized RCA and simplified LAMP (tRCA-lamp) gives rise to sufficient sensitivity and specificity in the detection of *V. parahaemolyticus* with a large background genome and has a high potential for versatile applications in the detection of pathogens in food and other complex samples.

## 2. Materials and Methods

### 2.1. Materials

All oligonucleotides used in this study were ordered from GENEWIZ (Suzhou, China), and their sequences are listed in Table S1. ATP, restriction endonucleases (*Hga*I and *Alu*I), and Bst 2.0 WarmStart DNA polymerase were purchased from New England Biolabs (Beijing, China). SYBR Green II, 96-well microtiter plates, 2 × DreamTaq PCR Master Mix, T4 DNA ligase, and the Thermo PCR product purification kit were purchased from Thermo Scientific (Pittsburgh, PA, USA). 2 × EvaGreen qPCR MasterMix was purchased from Applied Biological Materials Inc. (Richmond, BC, Canada). The bacterial genome extraction kit and marine organism genome extraction kit were purchased from Tiangen (Beijing, China). *Vibrio parahaemolyticus* (ATCC 17802) was provided by the Applied Microbiology Laboratory of Ocean University of China.

### 2.2. Activation and Cultivation of V. parahaemolyticus

*V. parahaemolyticus* (ATCC 17802) is preserved in glycerol at −80 °C in alkaline peptone water with 3% NaCl. An appropriate amount of *V. parahaemolyticus* was taken and cultured at 37 °C for 24 h in the LB liquid medium. After inoculating the initially activated strain again, it can be used for subsequent experimental studies.

### 2.3. Preparation of Artificially Contaminated Samples

The raw oyster samples purchased from the local supermarket (Qingdao, China) were tested by the traditional cultivation method and confirmed to be free from *V. parahaemolyticus*. The oysters of 25 g were added into 225 mL alkaline peptone water with 3% NaCl and smashed with a rotating blade homogenizer to prepare a 1:10 homogenate sample. Then, 0.5 mL bacteria solution was immediately added into 0.5 mL of the above oyster solution to obtain the artificially contaminated samples containing different bacteria numbers (e.g., for the sample of 200 cfu/g oysters, the 20 cfu/mL above bacterial solution is used). At the same time, sterilized 3% sodium chloride alkaline peptone water was used as blank control instead of a bacteria solution.

### 2.4. Genomic DNA Extraction from V. parahaemolyticus and Oyster

The bacterial genomic DNA (1 mL bacteria solution) was extracted with a bacterial genomic DNA extraction kit. The oyster genomic DNA (1 mL oyster solution or artificially contaminated samples) was extracted with a marine biological genomic DNA extraction kit. The extracted DNA was diluted by 50 μL TE buffer, and the concentration was determined by Nanodrop2000.

### 2.5. Purified Target Fragment Preparation by PCR

The reactions took place in a 10 μL system containing 10^5^ copies/μL Plasmid PUC 18, 0.4 μmol/L primers (EX-F-558 and EX-R-558 in Table S1), 1 × DreamTaq PCR Master Mix. The process includes an initial denaturation for 5 min at 95 °C and denaturation for 30 s at 95 °C, annealing for 30 s at 65 °C and extension for 1 min at 75 °C. After 30 PCR cycles, a step of extension for 5 min at 75 °C was added to assure the blunt ending of the double-stranded products (558 bp). The amplified products were purified using a Thermo PCR product purification kit.

### 2.6. Target Cyclization by Dynamic Adapter

Firstly, samples containing the target were digested by *Hga*I. The reactions took place in a 45 μL system containing 39.5 μL target samples, 5 U *Hga*I, 1 × NEBuffer1.1 (10 mmol/L Bis-Tris-Propane-HCl, 10 mmol/L MgCl_2_, 100 µg/mL BSA, pH 7.0@25 °C). The system was incubated at 37 °C for 1 h, and the *Hga*I was inactivated at 65 °C for 20 min. Then, the digested target was cyclized by dynamic adapters. The reactions took place in a 20 μL system containing 10 U T4 DNA ligase, *Hga*I digested products (10 μL), and dynamic adapters (10 nmol/L) in 1 × T4 DNA ligase buffer (500 μmol/L ATP, 10 mmol/L MgCl_2_, 10 mmol/L DTT, and 40 mmol/L Tris–HCl). The ligation was performed at 25 °C for 2 h and terminated by heating the mixture at 65 °C for 10 min.

The target cyclization was analyzed by quantitative PCR (qPCR). The reactions took place in a 10 μL system containing 1 μL target cyclization products, 0.4 μmol/L primers (F-558 and R-558 in Table S1), and 1 × EvaGreen qPCR Master Mix. The process included initial denaturation for 5 min at 95 °C and denaturation for 30 s at 95 °C, annealing for 30 s at 65 °C and extension for 1 min at 75 °C. After 30 PCR cycles, a step of extension for 5 min at 75 °C was added.

### 2.7. tRCA-Lamp Reaction

The amplification reactions took place in a 10 μL system containing 3.2 U Bst 2.0 WarmStart DNA Polymerase, 1.4 mmol/L dNTPs, 6 mmol/L MgSO_4_, 0.8 μmol/L FIP/BIP primers, 1 × SYBR Green II Fluorescent dyes, 5 μL cyclization product, and 1 × isothermal amplification buffer (20 mmol/L Tris-HCl (pH 8.8@25 °C), 50 mmol/L KCl, 10 mmol/L (NH_4_)_2_SO_4_, 2 mmol/L MgSO_4_, 0.1% Tween-20) in nuclease-free water. The amplification was performed at 65 °C for 50 min, and the fluorescence data were collected every minute.

### 2.8. T_m_ Simulation by Mfold

The *T*_m_ values of the adapters were simulated by The mfold Web Server using ‘Hybridization of two Different Strands of DNA or RNA’ [19]. The typical conditions are: [adapters] = 200 nmol/L, [Na^+^] = 10 mmol/L, [Mg^2+^] = 10 mmol/L.

## 3. Results

### 3.1. Strategy for the Combination of Target-Cyclized RCA and Simplified LAMP (tRCA-Lamp)

**Scheme 1:**
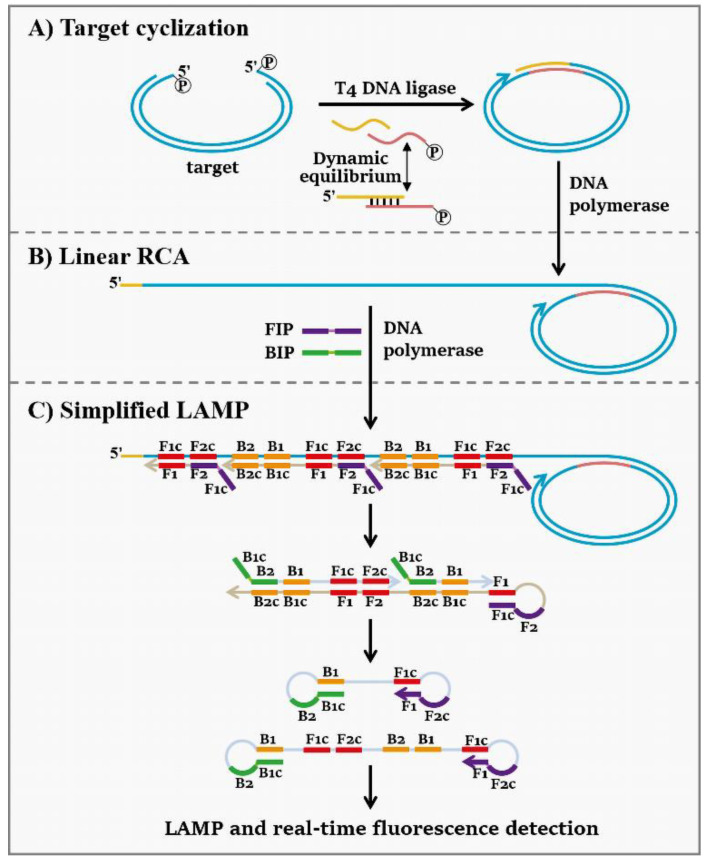
Illustration of the combination of target-cyclized RCA and simplified LAMP (tRCA-lamp). (**A**) Dynamic adapter cyclizes the target into a circular template. (**B**) Cyclized target is amplified to a long ssDNA with repeated sequences by linear RCA. (**C**) A simplified LAMP is carried out by only one pair of primers, and the amplified products can be detected by real-time fluorescence.

The principle of this method is composed of target cyclization and linear rolling circle amplification (tRCA) and simplified loop-mediated isothermal amplification (LAMP), which is schematically depicted in Scheme 1. Before target cyclization, a restriction enzyme (*Hga*I, $\begin{array}{c} 5^{\prime }\cdots GACGC{\left(N\right)}_{5}{}_{}^{▼}\cdots 3^{\prime }\\ 3^{\prime }\cdots CTGCG{\left(N\right)}_{10}{}_{▲}^{}\cdots 5^{\prime } \end{array}$) is used to digest DNA extracted from aquatic samples to fragments having sticky ends with random sequences. Then, a dynamic adapter with the sticky ends complementary to those of the target fragment cyclizes the target into a dsDNA ring. The dynamic here means the adapter is in an equilibrium state between hybridization and dissociation states because the *T*_m_ of its duplex part is close to or lower than the ligation temperature. Since one of the adapter’s 5′-ends is un-phosphorylated, a nick forms in the dsDNA ring to be used at the start site for primer extension in the subsequent RCA (Scheme 1A). The linear RCA and simplified LAMP perform simultaneously in the system. During the pre-amplification by linear RCA, the DNA ring serves as the template, making even trace targets convert to long ssDNA products with repeated sequences of hundreds of folds (Scheme 1B). Using these long repeated sequences of target DNA as the template for LAMP, hundreds of FIP primers can bind to one long molecule at the same time (Scheme 1C). With the processing of amplification, the product extended from the latter FIP displaces that from the former FIP, and an ssDNA with a loop at 5′ end forms. In the same way, BIP primers bind to the product from FIP and extend to the ssDNA with two loops, which is the core element of LAMP. In this way, only two primers (FIP and BIP) are able to carry out highly efficient LAMP, and the amplification products can be detected within tens of minutes.

Generally, isothermal DNA amplification is difficult to balance high sensitivity and high specificity in the presence of large amounts of background genome. Trace targets that are hidden in the sea of background genome are difficult to be captured, and some background sequences similar to the target are easily misidentified, resulting in non-specific amplification. For the tRCA-lamp system of this study, the sensitivity and specificity are separated and realized in two steps. This concept has been demonstrated in our previous papers [17]. During the tRCA, the dynamic adapter captures the target with high sensitivity due to the high cyclization efficiency, and the target is pre-amplified hundreds of times after linear RCA. During the simplified LAMP, the target sequences can be specifically and efficiently amplified because they have been pre-amplified by tRCA, and they are much more than those similar sequences in the background DNA. As a result, the interference from those similar sequences (easily causing non-specific amplification by non-specific primer binding) can be greatly reduced. Therefore, the primers identify the target sequences more easily and accurately so that LAMP performs with high specificity. Besides, the templates of LAMP are long repeated DNA sequences, so only one pair of primers are required, and the primer design is significantly simplified. Moreover, RCA and LAMP employ the same reaction conditions so that the amplification is achievable in one pot with isothermal mode. This method is applicable to any samples with large amounts of background genome.

### 3.2. High Sensitivity Capture and Cyclization of Target with Dynamic Adapters

**Scheme 2:**
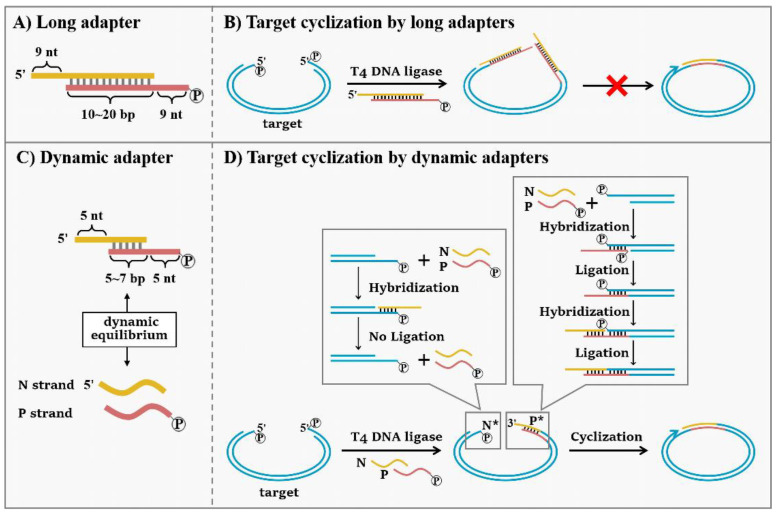
Molecular design of adapters for target cyclization. (**A**) The structure of long double-stranded adapters. (**B**) Target cyclization by adapters with the long duplex part. The adapters ligate on both sides of the target so that the cyclization cannot proceed. (**C**) The structure of short dynamic adapters. (**D**) Target cyclization by dynamic adapters. The short dynamic adapters ligate at the P* side first, followed by the ligation of another end (cyclization) to the N* side. No ligation between the adapter and the N* side can be carried out directly before the adapter ligated to the P* side. In this way, the cyclization becomes efficient, and the target can be amplified by RCA in the next step.

**Figure 1:**
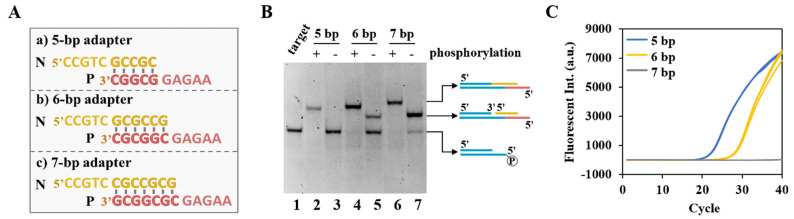
Ligation of DNA target by various adapters. (**A**) Sequences of the three adapters with various lengths. (**B**) DNA target (Ta/Tb) is ligated by adapters with 5–7 bp long duplex parts. Lane 1, the Ta/Tb duplex (38 bp) with a 5-nt overhang; lane 2, 4, 6, Ta/Tb is ligated by phosphorylated adapters; lane 3, 5, 7, Ta/Tb is ligated by un-phosphorylated adapters. [target DNA] = 1 μmol/L, [adapter] = 1 μmol/L, 2.5 U T4 DNA ligase at 25 °C for 2 h. (**C**) Cyclization products are analyzed by qPCR. Ligation reactions: [558-bp target] = 300 amol (10^7^ copies/μL), [adapter] = 10 nmol/L, 2.5 U T4 DNA Ligase at 25 °C for 2 h; qPCR reactions: [primers] = 0.4 μmol/L, 1 μL ligation products.

In the previous TC-RCA method we developed, a long double-stranded adapter (19-bp complementary section with two different 9-nt overhangs) was used to cyclize the target [17]. However, we found that the target cyclization yield was quite low (data not shown). The possible reason is that, for one target molecule, each of its ends can be easily ligated to one duplex adapter (two adapters for one target), resulting in failure of cyclization (Scheme 2A,B). To check whether this is true, we designed three types of adapters with 5-, 6- and 7-bp complementary parts, respectively (Figure 1A). For each type, an 5′-phosphorylated adapter (the strand **P** is phosphorylated, and strand **N** is un-phosphorylated) and un-phosphorylated adapter (either of the two strands is not phosphorylated) were used. A short duplex (38 bp, Ta and Tb in Table S1) with only one sticky end (phosphorylated) was used to mimic the target to simplify the reaction system. As shown in Figure 1B, when the duplex part is 7-bp long, efficient ligation (yield > 80%) was observed even when the adapter was not phosphorylated (Lane 7). The ligation yield was improved to 100% when the adapter was phosphorylated. This result indicates that each end of the target for cyclization can be ligated with a 7-bp adapter (as shown in Scheme 2B), respectively. It should be noted that the duplex part is only 7-bp long. Obviously, when the duplex is longer than 7 bp, this unexpected ligation can occur more easily because the ligation efficiency should be even higher. As a result, cyclization is prohibited.

In the case of the 6-bp adapter, when the un-phosphorylated adapter was used, the ligation yield decreased to about 40% (Lane 5), and the yield remained 100% for the phosphorylated one. In the case of the 5-bp adapter, however, no ligation product was observed for the un-phosphorylated adapter (lane 3), and the ligation yield also remained 100% for the phosphorylated one (lane 2). This indicates that the ligation efficiencies of short adapters are different between the phosphorylated (two sticky ends for ligation are phosphorylated) and un-phosphorylated form (one sticky end is phosphorylated, another is un-phosphorylated). In other words, the 5-bp adapter with only one strand 5′-phosphorylated can only ligate to one end of the target, and the form as shown in Scheme 2B will never happen. Interestingly, once this 5-bp adapter is ligated to one end of the target, the duplex part becomes extremely long and never dissociates under ligation conditions. Obviously, cyclization can occur efficiently because even the 7-bp adapter (un-phosphorylated) can ligate to Ta/Tb with a yield higher than 80% (lane 7). It should be noted that this cyclization is carried out by the ligation of sticky ends with 5-nt long overhangs, which is much more efficient for T4 DNA ligase [20,21].

The yields for target cyclization at low target concentrations (10^7^ copies/μL) using the above three adapters were also analyzed by quantitative PCR (qPCR). With the increase of the adapter lengths, as expected, the cyclization yield decreased gradually, and there are almost no cyclization products for the 7-bp adapter (grey lines, Figure 1C), indicating that adapters with long duplex parts are not conducive to target cyclization.

In detail, the new design of a short dynamic adapter can be described in Scheme 2C,D. The duplex part is 5 bp (for completely avoiding two adapters ligate to one target) or 6 bp (for improving the ligation efficiency of the adapter to one end of the target), and the overhangs are 5-nt long. The two complementary strands of the dynamic adapter are named **P** (red strand in Scheme 2C) and **N** (yellow strand in Scheme 2C), and the **P** strand is phosphorylated while the **N** strand is not. At the cyclization temperature (25 °C), the duplex parts of the adapters are unstable and in an equilibrium between hybridization and dissociation states. When the **N** strand binds to the sticky end of the target (N* side in Scheme 2D), it is unable to be ligated because the 5′-end of **N** has no phosphate group. When the **P** strand with phosphorylated 5′-end binds to the sticky ends of the target (P* side), it can ligate to the 3′-end of the P* side. After this ligation, the **N** strand can bind to the **P** strand with the help of T4 DNA ligase and is ligated to the 5′-end of P* side, forming a new sticky end. Therefore, only after both ends of the P* side ligate to the dynamic adapter the sticky end of the N* side can ligate to the newly born sticky end of the adapter (**N**/**P**), making the target cyclized to a double-stranded circular DNA with a nick. By using the dynamic adapter, there is almost no possibility that two double-stranded adapters ligate at both ends of the target, ensuring that most targets transform to the cyclized templates of RCA.

**Figure 2:**
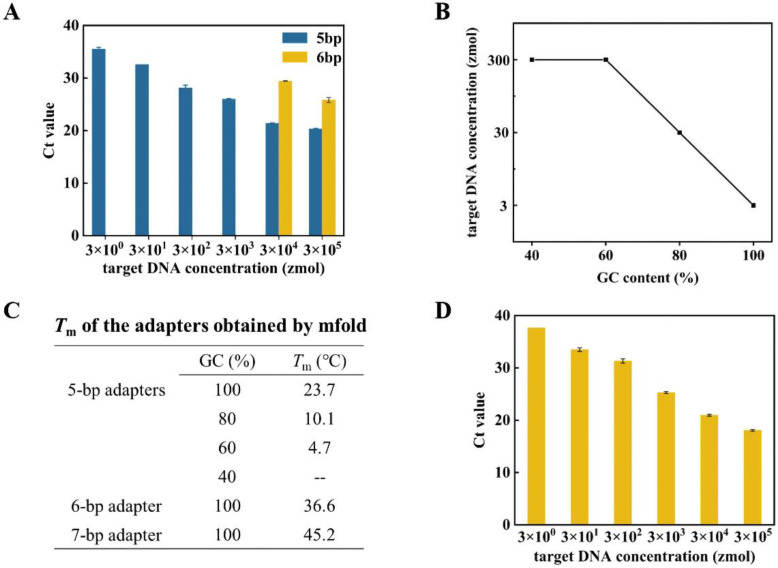
Target cyclization by dynamic adapters. (**A**) Target cyclization by 5-bp and 6-bp adapters with 100% GC content under various concentrations of targets. (**B**) The lowest target concentration that could be cyclized by 5-bp adapters with GC contents of duplex part from 40% to 100%. (**C**) *T*_m_ values of adapters simulated by Mfold under the conditions of 10 mmol/L Na^+^ and 10 mmol/L Mg^2+^. (**D**) Target cyclization by 5-bp adapter with 100% GC content in the presence of 300 ng oyster genome DNA. Ligation reactions: [adapter] = 10 nM, 2.5 U T4 DNA Ligase at 25 °C for 2 h; qPCR reactions: [primers] = 0.4 μmol/L, 1 μL ligation products, the C_t_ values were the average of three individual experiments.

In Figure 2A, the target was cyclized by 5-bp or 6-bp adapters with 100% GC content in their duplex parts, and the C_t_ values are used to evaluate the cyclization yield. With the increase of the duplex lengths of adapters, the cyclization efficiency decreased, which is consistent with the electrophoresis results (as shown in Figure 1B). For the 6-bp adapter, a target of fewer than 30 amol (10^6^ copies/μL) could not be cyclized by the adapter (the C_t_ value cannot be obtained). For the 5-bp adapter, the cyclization showed higher sensitivity which was achieved to 3 zmol (100 copies/μL) of the target. Accordingly, we used 5-bp adapters with various GC contents in the duplex part (40–100%). The results showed that the lowest target concentration that could be cyclized decreased (sensitivity of cyclization increased) greatly with the increase of GC content (Figure 2B). By analyzing the *T*_m_ values of adapters (Figure 2C), we found the *T*_m_ of the 5-bp adapter with 100% GC content is 23.7 °C, approaching the cyclization temperature (25 °C). The *T*_m_ of 6-bp (36.6 °C) and 7-bp (45.2 °C) adapter with 100% GC content is much higher. This indicates that only the 5-bp adapter with 100% GC content was at the well dynamic equilibrium between hybridization and dissociation states, and this equilibrium contributes to the high sensitivity of cyclization. When the GC content of the duplex part is in the range of 40–60% (*T*_m_ is lower than 25 °C), the sensitivity for cyclization decreases by about 100-fold.

When 300 ng of the oyster genome (3 × 10^3^ to 3 × 10^8^ folds more than the target) were added in the system as the background DNA, the 5-bp adapter with 100% GC still cyclized the target as low as 3 zmol (100 copies/μL) (Figure 2D), indicating that the influence of background genome on the target DNA detection is greatly suppressed by our newly designed dynamic adapter.

### 3.3. Anti-Interference Detection of V. parahaemolyticus by tRCA-Lamp

**Figure 3:**
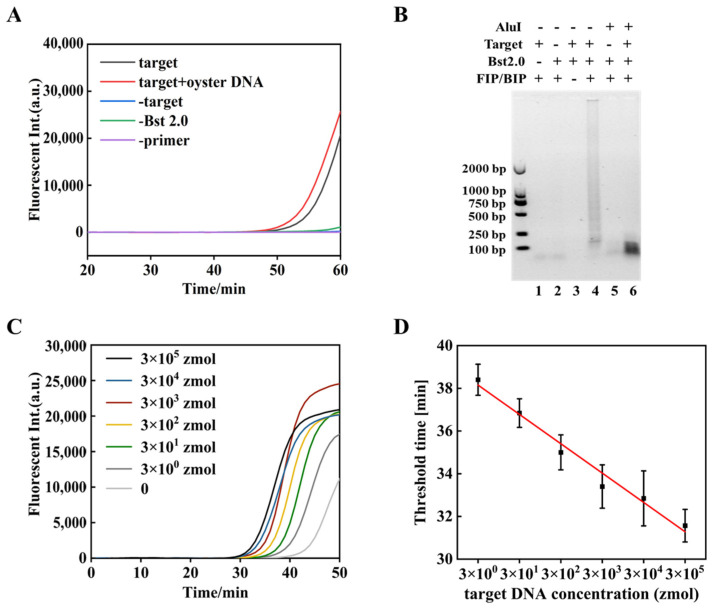
Detection of *V. parahaemolyticus* by tRCA-lamp. (**A**) Real-time fluorescence analysis. [target DNA] = 3 amol (10^5^ copies/μL), [FIP/BIP primers] = 1.6 μmol/L, [dNTP] = 1.4 mmol/L, 1 × SYBR Green II, 3.2 U Bst 2.0 DNA polymerase, 1 × isothermal amplification buffer at 65 °C for 60 min. (**B**) Endpoint product of tRCA-lamp with oyster DNA was digested with *Alu*I and analyzed on 1% agarose gel electrophoresis. Lane 1–3, negative controls; lane 4, the product of tRCA-lamp; lane 5, 1 U *Alu*I was added to the sample of lane 2; lane 6, 1 U *Alu*I was added to the sample of lane 4. (**C**) Fluorescent intensity of tRCA-lamp under optimized conditions. [dNTPs] = 1.4 mmol/L, [primers] = 0.8 μmol/L, [MgSO_4_] = 6 mmol/L, 3.2 U Bst 2.0 DNA Polymerase, 1 × isothermal amplification buffer at 65 °C for 50 min. (**D**) Threshold time (T_t_) was plotted against the lg[target concentration] of the reaction.

In this part, *V. parahaemolyticus* contamination of oyster genome was detected by our newly developed method, as shown in Scheme 1. The target sequence (605 bp) is in the maltoporin precursor gene (GeneBank number: JF747207.1), which has been determined as the intraspecies conserved sequence of *V. parahaemolyticus* [22]. The primer sequences of LAMP (FIP and BIP) are shown in Table S1. Because only two primers are used, the non-specific amplification of LAMP is expected to be greatly suppressed. The reactions involving RCA and LAMP are achievable in one pot in an isothermal model. The commonly used SYBR GREEN II was used to stain the DNA duplex products. Only in the presence of the cyclized target were the amplification reactions triggered, and the fluorescent intensity rapidly increased. Typical results of tRCA-lamp for the detection of *V. parahaemolyticus* are shown in Figure 3A.

The result showed that the fluorescence signal was efficiently amplified in the presence of the 3 amol target (10^5^ copies/μL) within 60 min (black line in Figure 3A). When the oyster genome was added, the systems with the background genome (red line) had similar amplification efficiency with the purified bacteria. This indicates that the background genome has extremely little effect on the target cyclization and amplification. It should be noted that the 3 × 10^5^ folds of background DNA usually decreases greatly both the sensitivity and specificity. The fact that no decrease of sensitivity was observed indicates that this decrease was counteracted by the sensitivity improvement of our approach [17]. To verify the specificity of this approach, the amplification products with the oyster genome are digested by restriction enzyme (*Alu*I, $\begin{array}{c} 5^{\prime }\cdots AG_{}^{▼}CT\cdots 3^{\prime }\\ 3^{\prime }\cdots TC_{▲}^{}GA\cdots 5^{\prime } \end{array}$). The results in Figure 3B show that all the amplification products were digested into short fragments with expected length, indicating that no non-specific amplification occurred.

### 3.4. Sensitivity of the tRCA-Lamp

After the optimization of the reaction (Figure S1), the sensitivity of tRCA-lamp was analyzed in oyster genome under the optimal conditions (0.8 μmol/L primers, 1.4 mmol/L dNTP, 6 mmol/L MgSO_4_ at 65 °C). Figure 3C show that the fluorescence signal was efficiently amplified within 50 min when the target is over a range of 3 zmol–300 amol (10^2^–10^7^ copies/μL), and the target was clearly detected down to as low as 3 zmol. In order to quantitatively analyze the efficiency of tRCA-lamp, threshold time (T_t_: the time when the fluorescent intensity exceeds an arbitrary threshold) was defined in analogy to threshold cycle (C_t_) in real-time PCR analysis [23]. Obviously, the lower T_t_ values indicate the higher amplification efficiency. The T_t_ values linearly increased with the logarithm of [target DNA] over the range of 3 zmol–300 amol. The linear equation is y = 41.024 − 1.4343x (R^2^ = 0.9874) (Figure 3D).

### 3.5. Fluorescence and Visual Detection of Artificially Contaminated Oyster Samples

**Figure 4:**
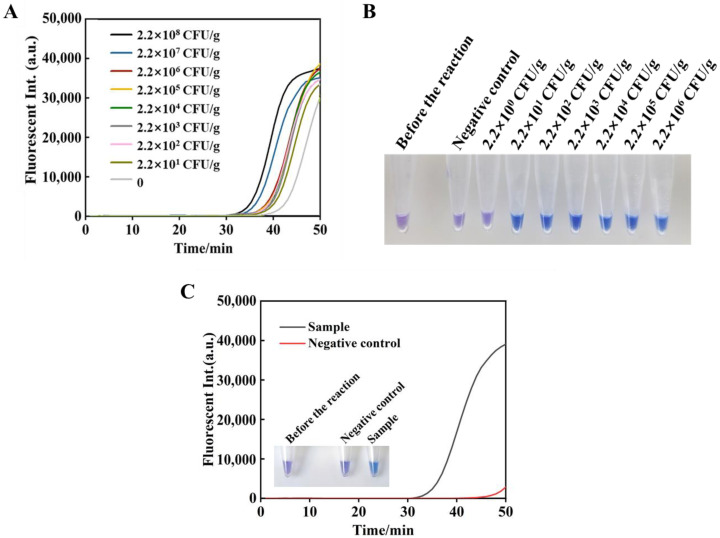
Target detection for artificially contaminated oyster samples. (**A**) Real-time fluorescence analysis. [dNTPs] = 1.4 mmol/L, [primers] = 0.8 μmol/L, [MgSO_4_] = 6 mmol/L, 3.2 U Bst 2.0 DNA Polymerase, 1 × isothermal amplification buffer at 65 °C for 50 min. (**B**) Visual detection of *V. parahaemolyticus* by hydroxynaphthol blue (HNB). [HNB] = 120 μmol/L, amplification time is 40 min. (**C**) Detection result of the unknown sample.

Herein, tRCA-lamp is used for the detection of oyster samples artificially contaminated by *V. parahaemolyticus* over a range of 2.2 × 10^1^–2.2 × 10^8^ cfu/g (Figure 4). The fluorescence result showed that all the amplification curves are clearly distinguished from the negative control (light grey line in Figure 4A), and the amplification efficiency grows gradually with the increase of target concentrations. However, the linear relationship between target concentrations and the T_t_ values is not so perfect in the artificially contaminated samples. The possible reason is that DNA extraction is not so efficient, and the residual protein and metal ions in the extracted DNA may also affect the amplification. Therefore, the DNA extraction process should be optimized further in later studies.

Visual detection can make the results more intuitive and convenient without using a fluorescence detector. Here, visual detection was realized by adding hydroxynaphthol blue dye (HNB) in the amplification system (Figure 4B) [24]. Before the amplification, HNB is purple due to its binding with Mg^2+^. With the proceeding of amplification, Mg^2+^ forms sediment gradually with the pyrophosphates so that HNB becomes blue, its original colour (Figure S2). The colour change with target concentration was observed as expected. By optimizing HNB concentration (120 μmol/L) and reaction time (40 min) after adding HNB (Figure S3), samples for 2.2 × 10^1^–2.2 × 10^6^ cfu/g of the target changed from purple to blue (Figure 4B), indicating that HNB can clearly distinguish the difference in target concentration of *V. parahaemolyticus*. Besides, an unknown sample is detected by this method (Figure 4C), and it can be clearly distinguished with the negative control in both fluorescence analysis and visual detection. The T_t_ value of this sample is 34.39, and the concentration of *V. parahaemolyticus* is calculated as about 10^8^ cfu/g, which is consistent with the plate count (0.6 × 10^8^ cfu/g).

## 4. Discussion

An isothermal pathogen detection method was accomplished with the combination of target cyclization, linear RCA, and LAMP (tRCA-lamp) and showed high sensitivity and specificity. In our present method, a novel kind of dynamic adapter is used, and the ligation activity of its two ends is quite different due to phosphorylation, which ensures one end ligating to the adapter first, and the other end later. By this design, the cyclization efficiency was greatly improved by avoiding both ends of the target fragment attaching to the adapter, respectively. For the previous 19-bp adapter, it can form a very stable double strand under reaction conditions so that both terminals are present in stable sticky ends. In addition, the adapters are usually used with a much higher concentration than that of target DNA. As a result, each end of the target is ligated with one duplex adapter (two adapters for one target) so that the efficiency of cyclization is extremely low. For the present 5-bp dynamic adapter, whose *T*_m_ value is even lower than the ligation temperature, it is in an equilibrium state between single-stranded (dissociation) and duplex (hybridization) state. This temporary duplex formation is necessary for efficient cyclization because lower GC content (40–60%) of the duplex part in the adapter caused much lower cyclization sensitivity than that of 100% GC content. Not only the *T*_m_ of the duplex part but also its length (5 bp) is essential. When the duplex part is 6 bp long (66% GC content), and its *T*_m_ (25.7 °C) is close to the reaction temperature (25 °C), the sensitivity is only about 30 amol (10^6^ copies/μL) (Figure S4), indicating that even the un-phosphorylated adapter can ligate to the sticky end of the target. The ligation efficiency of this dynamic adapter can be explained by the characteristics of T4 DNA ligase. For efficient ligation of a nick, 5–6 bp length on both sides of the nick is essential [25,26]. At the N* side, the **P** strand (with less ligation efficiency than that at the P* side due to the un-phosphorylation of the **N** strand) cannot ligate to the target because the **P** strand dissociates from the **N** strand before the ligation (Scheme 2). Accordingly, by using the exact 5-bp adapter with 100% GC content almost all targets can be cyclized to form circular template for the subsequent linear RCA, and the detection sensitivity is significantly improved. It should be mentioned that the length of the target gene is better to be longer than 200 bp. For short genes, the duplex structure is difficult to bend due to lack of flexibility and cannot realize highly efficient cyclization [17].

This method also showed high anti-interference ability against large amounts of background DNA. In previous RCA methods, padlock probes directly captured trace targets in large amounts of background DNA [27], making the possible occurrence of non-specific binding. In our present method, *V. parahaemolyticus* genome and background nucleic acids (oyster genome in this paper) are firstly digested by the restriction enzyme (*Hga*I) to fragments whose overhangs are 5-nt random sequences. The target fragment is cyclized by the adapter whose sticky ends are complementary to the target. In the step of linear RCA, these cyclized targets are amplified hundreds to thousands of times and “stand out” among a large amount of background DNA. Then, the primers of LAMP recognize the target sequence more easily and more accurately so that non-specific amplification can be greatly suppressed. Of course, it is possible that some accompanying fragments digested from background DNA are also cyclized by the adapter, but these fragments cannot interfere with the detection due to the following reasons. On the one hand, the probability that fragments from the background DNA have the same two sticky ends as the target is only 1/10^9^, and there is less than one such fragment in every oyster genome (~800 Mb) [28,29]. On the other hand, although these accompanying fragments can be cyclized and pre-amplified by linear RCA, their RCA products cannot be recognized by the LAMP primers and will not influence the specificity of the detection.

The combination of RCA and LAMP showed the potential for high efficiency. The previous TC-RCA approaches developed by our team used hyper-branched rolling circle amplification (HRCA) to amplify the cyclized target, and the reaction time was up to 16 h [17]. Our present method uses RCA-LAMP to amplify the cyclized target, and the reaction times were greatly shortened to 40 min. Tian et al. designed an amplification method using padlock-RCA and LAMP and detected miRNA within 80 min [30]. The main difference between this method and ours is that this method needs to design primers with a hairpin to form the intermediate products with two loops, which are the key elements for LAMP. The primers for our method directly target the gene sequence of *V. parahaemolyticus* and can trigger the complete LAMP without special design (can directly use the primer design software of LAMP), making the primer design greatly simplified. The detailed principle of the simplified LAMP is shown in Figure S5.

## 5. Conclusions

Using a dynamic adapter and combining RCA and LAMP, a new nucleic acids amplification strategy (target-cyclized rolling circle amplification with loop-mediated isothermal amplification, tRCA-lamp) was developed with high efficiency, sensitivity, and specificity. RCA and LAMP have the same reaction conditions and can realize one-pot amplification. Besides, visual detection was realized by adding HNB to the system. This method detects *V. parahaemolyticus* with a detection limit of 22 cfu/g. We believe that the tRCA-lamp method can provide a new path for the quantitative and timely molecular testing of various pathogens in food and other complex environmental samples. A detection kit based on this method is currently under development in our laboratory.

## Data Availability

Not applicable.

## Supplementary Materials

The following supporting information can be downloaded at: https://www.mdpi.com/article/10.3390/foods11030352/s1, Table S1: Sequences of oligonucleotides used in this study; Figure S1: Optimization of reaction conditions for tRCA-lamp; Figure S2: Principle of the colour changes of HNB before and after the amplification; Figure S3: Optimization of the visual detection by HNB; Figure S4: Target cyclization by 6-bp adapters with GC contents of duplex part from 50% to 100%; Figure S5: Detailed principle of simplified LAMP.
