# Supplementary material for: Anti-Interference Detection of Vibrio parahaemolyticus from Aquatic Food Based on Target-Cyclized RCA with Dynamic Adapter Followed by LAMP
Source: Foods. 2022 Jan 26;11(3):352. doi: 10.3390/foods11030352 (PMC8834026; doi:10.3390/foods11030352)
Supplement: Supplementary file 1 [file foods-11-00352-s001.zip › foods-1559027-supplementary.pdf]

Table S1. Sequences of oligonucleotides used in this study.

Name	sequence (5′-3′)	nt
**EX-F-558**	ACTCAACCAAGTCATTCTGAGA	22
**EX-R-558**	TCTGATGCCGCATAGTTAAGC	21
**F-558**	AATAAGGGCGACACGGAAAT	20
**R-558**	TGGATCTCAACAGCGGTAAG	20
**Ta**	ACTTCTGACAACGATCGGAGGACCGAAGGAGCTAACCG	38
**Tb**	GACGGCGGTTAGCTCCTTCGGTCCTCCGATCGTTGTCAGAAGT	43
**558-5-5N**	CCGTC**GCCGC**	10
**558-5-5P**	AAGAG**GCGGC**	10
**558-5-4N**	CCGTC**GCTGC**	10
**558-5-4P**	AAGAG**GCAGC**	10
**558-5-3N**	CCGTC**GTCAC**	10
**558-5-3P**	AAGAG**GTGAC**	10
**558-5-2N**	CCGTC**GTTAC**	10
**558-5-2P**	AAGAG**GTAAC**	10
**558-6-6N**	CCGTC**GCGCCG**	11
**558-6-6P**	AAGAG**CGGCGC**	11
**558-7-7N**	CCGTC**CGCCGCG**	12
**558-7-7P**	AAGAG**CGCGGCG**	12
**605-5-5N**	TAGTT**GCCGC**	10
**605-5-5P**	CGCTA**GCGGC**	10
**FIP**	TCGTTCCATTTGTACATTGGACGTACAACTAAGTACGACTCTAGC	45
**BIP**	CTATGCGTACTATTTTTGAAGCTGGCACTGTTAGCACCACCGAAGT CA	48

Note: a) **EX-F-558** and **EX-R-558** are the primers to prepare the target used in target cyclization research; b) **F-558** and **R-558** are the primers to detect the target cyclization yield by qPCR; **c) Ta** and **Tb** form the short duplex to mimic the target in Figure 1A; d) **558-5-5N** to **558-7-7P** are the sequences of dynamic adapters in target cyclization research. The sequence **N** is un-phosphorylated strand and the sequence **P** is phosphorylated strand. The bold parts are the complimentary parts of the adapters; e) **605-5-5N** and **605-5-5P** are the sequences of the adapter used in the detection of *V. parahaemolyticus*; f) **FIP** and **BIP** are the primers for simplified LAMP; g) The numbers of 558 and 605 indicate the length of the DNA target fragments.

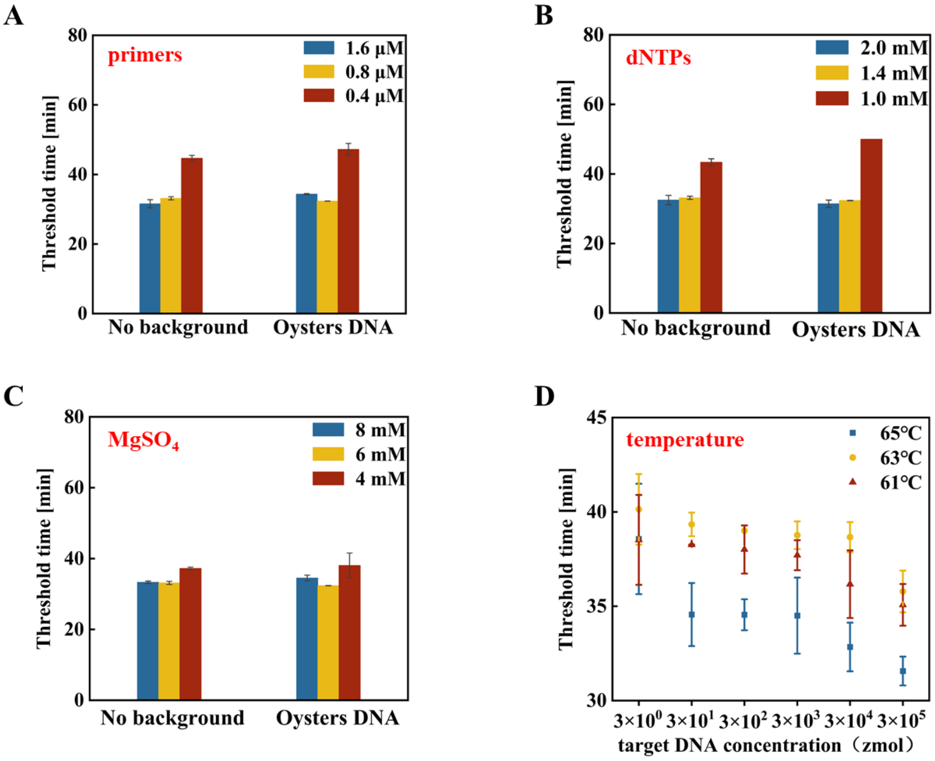

Figure S1. Optimization of reaction conditions for tRCA-lamp. (**A**) Concentration of primer. [target DNA] = 3 amol, [$MgSO_4$] = 8 mmol/L, [dNTP] = 1.4 mmol/L and 3.2 U Bst2.0 DNA polymerase at 65°C for 1 h. (**B**) Concentration of dNTP. [target DNA] = 3 amol, [primers] = 0.8 μmol/L, [$MgSO_4$] = 8 mmol/L and 3.2 U Bst2.0 DNA polymerase at 65°C for 1 h. (**C**) Concentration of $MgSO_4$. [target DNA] = 3 amol, [primers] = 0.8 μmol/L, [dNTP] = 1.4 mmol/L and 3.2 U Bst2.0 DNA polymerase at 65°C for 1 h. (**D**) Reaction temperature. [target DNA] = 3 amol, [primers] = 0.8 μmol/L, [$MgSO_4$] = 8 mmol/L, [dNTP] = 1.4 mmol/L and 3.2 U Bst2.0 DNA polymerase for 1 h.

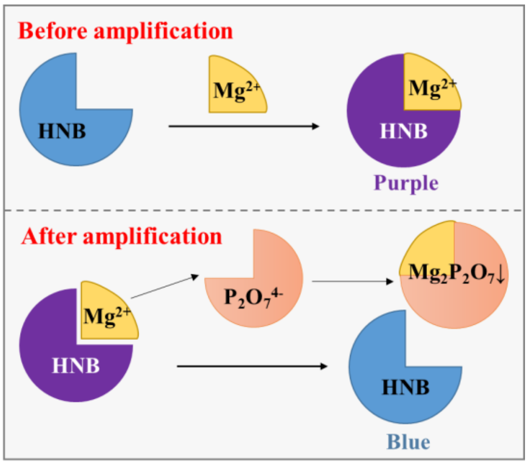

Figure S2. Principle of the colour changes of HNB before and after the amplification. Before the amplification, HNB is purple due to its binding with Mg^{2+}. With the proceeding of amplification, Mg^{2+} forms sediment gradually with the pyrophosphates so that HNB becomes blue, its original colour.

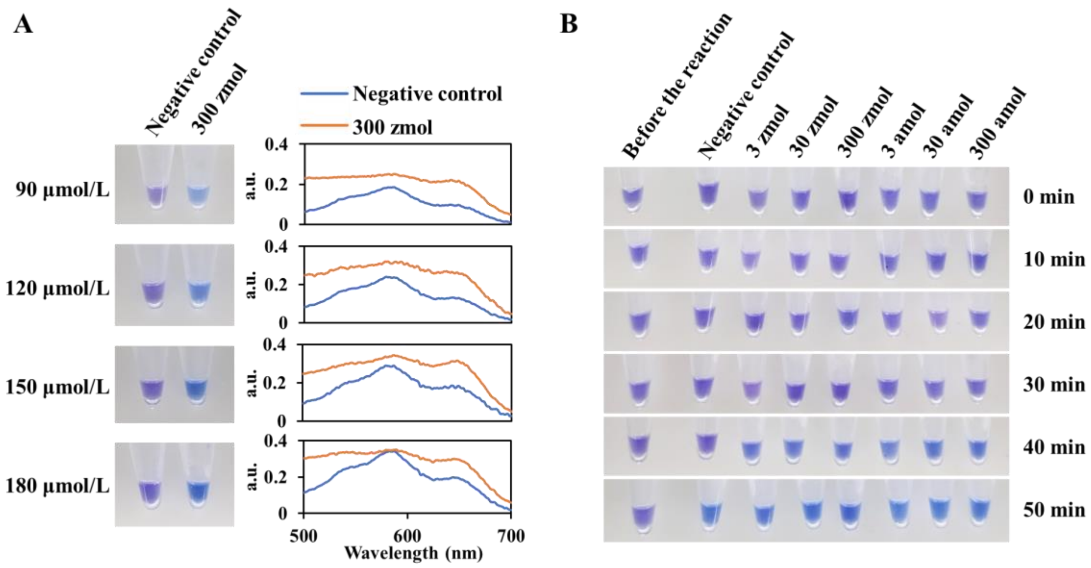

Figure S3. Optimization of the visual detection by HNB. (**A**) Colour changes under various concentrations of HNB. The corresponding spectra are also shown. Amplification time is 40 min. (**B**) Colour changes under different amplification time. [HNB]=120 µmol/L.

A

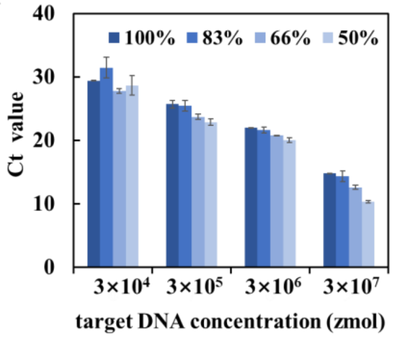

B

T_m of 6-bp adapters obtained by mfold

GC (%)	T_m (°C)
100	36.6
83	29.2
66	25.7
50	20.3

Figure S4. Target cyclization by 6-bp adapters with GC contents of duplex part from 50% to 100%. (**A**) Ligation reactions: [adapter] = 10 nmol/L, 2.5 U T4 DNA Ligase at 25°C for 2 h; qPCR reactions: [primers] = 0.4 μmol/L, 1 μL ligation products, the Ct values were the average of three individual experiments. (**B**) T_m values of adapters simulated by Mfold under the conditions of 10 mmol/L Na^+ and 10 mmol/L Mg^{2+}.

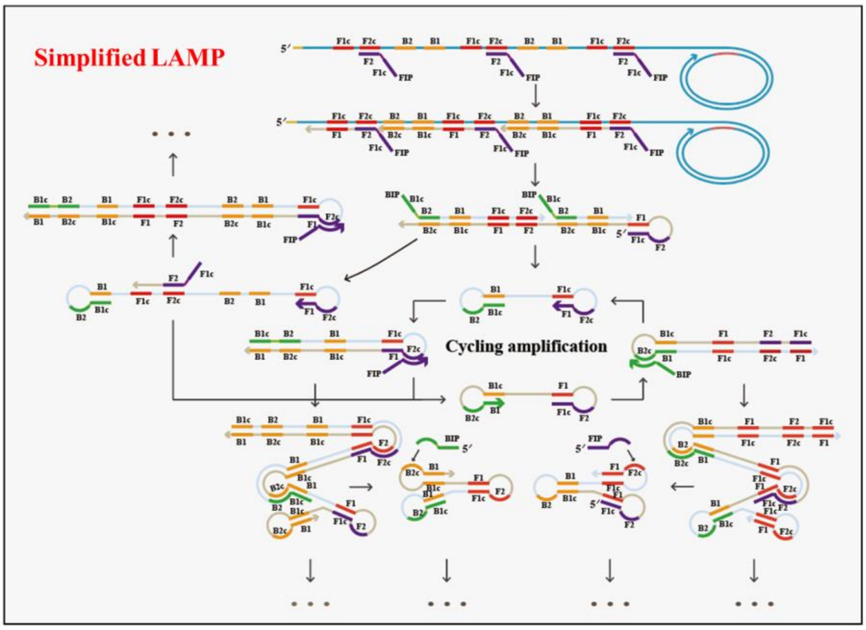

Figure S5. Detailed principle of simplified LAMP.
